# Supporting public health communities worldwide

**DOI:** 10.1186/s44263-024-00051-8

**Published:** 2024-03-13

**Authors:** Ben Cranfield, Gen Li

**Affiliations:** 1 BioMed Central, Springer Nature, London, UK; 2 BioMed Central, Springer Nature, Shanghai, China

*The challenges and opportunities in supporting global health are increasingly diverse as they are important. In order to publish timely, high-quality and equitable research that promotes health and well-being for everyone, BMC Global and Public Health aims to develop an expansive portfolio of content and targeted collections.*

We are excited about the potential impact that *BMC Global and Public Health* will have in promoting sound science and debate in the pursuit of global health equity. The journal opened with its first publications in July 2023, including the launch editorial that introduced the journal’s aims and ambitions [1]. Our intention is to publish content from a wide range of communities to encourage health benefits for all. *BMC Global and Public Health* is now steadily diversifying across research fields and starting to engage with communities across the globe.

Together, the Editorial team has a wide range of public health interests. Ben has a background in primary care clinical trials and his PhD research focused on general practitioners use of blood tests for suspected cancer patients. Cancer continues to be a global epidemic where primary care remains an important setting for targeting prevention and diagnostic strategies. However, opportunities for early cancer detection vary by geography, whereby factors relating to healthcare structures, resources, and access can determine cancer outcomes [2, 3]. To support communities where improved strategies are needed for supporting earlier cancer diagnosis, we launched the collection *Implementation of cancer strategies in primary care: Addressing global disparities* (https://www.biomedcentral.com/collections/ICSPC) - guest edited by Tanimola Martins (University of Exeter) and Suzanne Scott (Queen Mary University of London). This year’s World Cancer Day focused on closing the care gap—we hope this collection will contribute towards this goal by attracting valuable content that helps to address global cancer inequalities and promote strategies for earlier detection. Equality in healthcare is also dependent on the spread and advance of technology. We recognize the opportunities for novel and existing technologies to reduce barriers to accessing healthcare and promote sustainable solutions. To capture impactful content in this field, we launched the collection *Digital interventions, services, and applications for reducing global health gaps in disadvantaged populations* (https://www.biomedcentral.com/collections/DIGH) - guest edited by Dustin Gibson (Johns Hopkins University) and Binyam Tilahun (University of Gondar). An influx in health technology and pharmaceutical advances in recent years may in part be associated with the emergence of COVID-19. The pandemic has reinvigorated the infectious disease research community’s focus on preparedness and control measures, leading to a growing interest in travel-related infectious disease. This is an important and developing area of research where we hope our collection *Epidemiology of travel-related and tropical infectious diseases* (https://www.biomedcentral.com/collections/etid) - guest edited by Asma Sohail (Monash University) and Shanti Narayanasamy (Duke University) - will stimulate ongoing scientific discourse.

Gen is interested in mental health-related topics including treatment, social determinants, and public health service for mental disorders. He has research experience in epidemiology and intervention studies of common mental disorders such as depression and posttraumatic stress disorder. Mental health is as important as physical health and there is no health without mental health. It is always important to introduce a psychosocial perspective to gain a better understanding of current public health challenges faced by vulnerable populations. Therefore, we launched the collection *Stigma and mental health in infectious diseases* (https://www.biomedcentral.com/collections/stigma-mh-infectious). We believe that health-related stigma and mental health problems constitute major barriers to help seeking behavior and high-quality health services, especially for infectious diseases such as HIV and tuberculosis. In this collection guest edited by Amrita Daftary (York University) and Jeremiah Chikovore (Human Sciences Research Council, South Africa), we aim to discuss the assessment, health impacts, and intervention of stigma and common mental disorders associated with infectious diseases, and the first content has already been published. In their research, Cooper et al. explored race/ethnicity-based stigmatization in Canada during the COVID-19 pandemic [4]. In a Q&A with the guest editors, their experience and perspectives regarding pressing issues in addressing health-related stigma and mental health problems were also shared [5].

Universal health coverage (UHC), as an important aim of the Sustainable Development Goal 3 (Ensure healthy lives and promote well-being for all at all ages), is another key topic for the journal. Migration health is a key puzzle to achieving UHC. In October 2023, the World Health Organization published its first global research agenda on migration health, which prioritized the generation of evidence in primary health care among migrants and refugees [6]. Therefore, we launched the collection *Making health care more accessible, acceptable, and sustainable to migrants and refugees* (https://www.biomedcentral.com/collections/MHCAS), guest edited by Wen Chen (Sun Yat-sen University). In this collection, we aim to present evidence from multi-sectoral research in this underrepresented population to address determinants of health. Major improvements in health financing are also required to achieve meaningful progress in UHC. Unwise government spending on healthcare would even exacerbate existing health inequity. We therefore launched the collection *Health financing to advance universal health coverage and health equity – challenges and novel approaches* (https://www.biomedcentral.com/collections/HF), guest edited by Samir Garg (State Health Resource Centre Chhattisgarh), to capture global experiences and novel approaches in health financing research. With these collections, we hope to capture evidence that helps to inform policy and practice and encourages the transition to UHC by 2030.

Global health remains an important research and public priority. We are delighted to serve public health communities around the world by helping researchers to distribute valuable evidence and by engaging with healthcare advocates. With support from all our stakeholders including the guest editors for our collections, *BMC Global and Public Health* aspires to be a leading journal that contributes to advancing research evidence and to improving human health.

## Data Availability

Not applicable.

## References

[1] John-Schuster G. Introducing BMC Global and Public Health. BMC Glob Public Health. 2023;1:1. 10.1186/s44263-023-00012-7. 10.1186/s44263-023-00012-7 PMC11622847 39681870

[2] Rubin G, Berendsen A, Crawford SM, Dommett R, Earle C, Emery J, Fahey T, Grassi L, Grunfeld E, Gupta S, Hamilton W, Hiom S, Hunter D, Lyratzopoulos G, Macleod U, Mason R, Mitchell G, Neal RD, Peake M, Roland M, Seifert B, Sisler J, Sussman J, Taplin S, Vedsted P, Voruganti T, Walter F, Wardle J, Watson E, Weller D, Wender R, Whelan J, Whitlock J, Wilkinson C, de Wit N, Zimmermann C. The expanding role of primary care in cancer control. Lancet Oncol. 2015;16(12):1231–72. 10.1016/S1470-2045(15)00205-3. PMID: 26431866. 26431866 10.1016/S1470-2045(15)00205-3

[3] Omotoso O, Teibo JO, Atiba FA, Oladimeji T, Paimo OK, Ataya FS, Batiha GE, Alexiou A. Addressing cancer care inequities in sub-Saharan Africa: current challenges and proposed solutions. Int J Equity Health. 2023;22(1):189. 10.1186/s12939-023-01962-y. PMID: 37697315; PMCID: PMC10496173. 37697315 10.1186/s12939-023-01962-y PMC10496173

[4] Cooper J, Theivendrampillai S, Lee T, et al. Exploring perceptions and experiences of stigma in Canada during the COVID-19 pandemic: a qualitative study. BMC Glob Public Health. 2023;1:26. 10.1186/s44263-023-00020-7. 38798820 10.1186/s44263-023-00020-7 PMC11116254

[5] Daftary A, Chikovore J. Stigma and mental health in infectious diseases—meet the guest editors. BMC Global Public Health. 2024;2:11. 10.1186/s44263-024-00041-w.

[6] Global research agenda on health. migration and displacement: strengthening research and translating research priorities into policy and practice. Geneva: World Health Organization; 2023.

